# The Somatotopy of Mental Tactile Imagery

**DOI:** 10.3389/fnhum.2019.00010

**Published:** 2019-02-18

**Authors:** Timo Torsten Schmidt, Felix Blankenburg

**Affiliations:** Neurocomputation and Neuroimaging Unit, Department of Education and Psychology, Freie Universität Berlin, Berlin, Germany

**Keywords:** tactile, mental imagery, imagery debate, tactospatial sketchpad, somatosensory, fMRI, mental codes

## Abstract

To what degree mental imagery (MI) bears on the same neuronal processes as perception has been a central question in the neurophysiological study of imagery. Sensory-recruitment models suggest that imagery of sensory material heavily relies on the involvement of sensory cortices. Empirical evidence mainly stems from the study of visual imagery and suggests that it depends on the mentally imagined material whether hierarchically lower regions are recruited. However, evidence from other modalities is necessary to infer generalized principles. In this fMRI study we used the somatotopic organization of the primary somatosensory cortex (SI) to test in how far MI of tactile sensations activates topographically sensory brain areas. Participants (*N* = 19) either perceived or imagined vibrotactile stimuli on their left or right thumbs or big toes. The direct comparison to a corresponding perception condition revealed that SI was somatotopically recruited during imagery. While stimulus driven bottom-up processing induced activity throughout all SI subareas, i.e., BA1, BA3a, BA3b, and BA2 defined by probabilistic cytoarchitectonic maps, top-down recruitment during imagery was limited to the hierarchically highest subarea BA2.

## Introduction

The human ability to mentally represent and manipulate information in the absence of sensory stimulation is key for any higher cognitive functions. Neuroscientific research on mental imagery (MI) addresses the question of how our brains generate and represent mental content. Critically, most research stems from studies in the visual modality, leaving open the question of whether findings, models and theories generalize to other modalities.

The so called *imagery debate* was focused on the question what type of code our brains use to represent mental content (Pearson and Kosslyn, 2015). In particular, the controversial issue was discussed whether besides *symbolic* (also termed language-dependent, categorical, conceptual, or non-sensory) also *depictive* (also called pictorial, non-language dependent, sensory, or non-conceptual) codes are to be found in the brain. Acclaimed psychological experiments have provided evidence that mere processing of rules and symbols cannot account for higher human cognition. Most famously Shepard and Metzler (1971) demonstrated that mental rotation relies on information represented in *analog form* as they found reaction times to linearly increase with the angle of a 3D-rotation that participants mentally performed. Furthermore, Kosslyn carried out a series of imagery experiments where participants had to *scan* a mental image to perform spatial judgments (e.g., the *mental island-walk* experiment; Kosslyn et al., 1978). He argued that mental representations directly reflect physical stimulus properties. In particular, mental representations of spatial layouts are proposed to be based on the actual distance of the real object. Hence, mental images are not represented in terms of propositional logic. Instead, they are directly linked to perceptual processes, which automatically code isomorphic properties in the same way that they usually process information obtained from the senses.

Neuroimaging work has emphasized the role of perceptual processing in sensory cortices during MI. Particularly the retinotopic organization of visual cortices is thought to implement spatial features of mental images and thereby support *depictive* codes in the brain. Indeed, retinotopic activation of visual cortices during MI has been reported and related to the representation of spatial features of mental images (Klein et al., 2004; Slotnick et al., 2005; Naselaris et al., 2015). With the rise of positron emission tomography, imagery research started to test for the recruitment of perceptual regions (reviewed in Kosslyn, 2005). This hypothesis has been the preeminent view in the imaging literature with a heavy focus of functional magnetic resonance imaging (fMRI) studies (a meta-analysis can be found in McNorgan, 2012; a recent review with links to clinical studies in Pearson et al., 2015) and current neuroimaging studies support the importance of perceptual processes for MI (Cichy et al., 2012).

In comparison to vision, the somatosensory system is characterized by fewer low-level processing steps (Felleman and Van Essen, 1991). Meanwhile, stimulus features such as spatial arrangements, orientations, shape, or intensity appear similarly processed as in vision (Sripati and Bensmaia, 2006; Bensmaia et al., 2008). Particularly the somatotopic organization (in parallel to retinotopy) of the primary somatosensory cortex (SI) allows testing for activation that is specific to the body location of tactile imagery. SI is constituted of the four subregions BA3a, BA3b, BA1, and BA2 that are arranged across the postcentral gyrus. While BA3a (as well as BA2 to a lesser extent) mainly processes proprioceptive signals, the thalamic afferents submitting signals from cutaneous receptors innervate BA3b, BA1, and BA2 (Kaas, 1993; Grefkes et al., 2001). Their anatomic anterior-posterior gradient is also reflected in their hierarchical order with BA2 as the highest subregion within SI (Felleman and Van Essen, 1991).

Human neuroimaging work on tactile MI is very limited. McNorgan (2012) summarized fMRI studies from different modalities and identified only three tactile studies at that time. In two of these studies, the participants had to imagine somewhat complex properties, e.g., performing a sensory action or haptic/form judgments (Newman et al., 2005; Olivetti Belardinelli et al., 2009). A study by Yoo et al. (2003) was the only one that directly compared a perception condition with an imagery condition. They used brushing stimuli and reported time-course data from SI and SII. These showed signal increases in both regions related to imagery. In Schmidt et al. (2014), we have reported further evidence for sensory recruitment in the somatosensory system. Participants performed a spatial judgment task for a Braille-like vibrotactile stimulus or a mental image of such. A direct comparison between perception and imagery revealed an overlapping activation in the finger-region of SI. Finally, De Borst and De Gelder (2017) used a multivariate whole-brain searchlight approach in a paradigm with haptically explored figures and reported support for overlapping neuronal codes in sensory regions between perception and imagery.

In the fMRI study at hand the perception of tactile stimuli at four different body locations (left/right hands/feet) is compared to MI of corresponding stimulation. We applied simple vibratory stimuli to minimize high-order feature processing and used stimuli without spatial layout information to focus on somatotopic processing only. We first aimed to test for the network of brain regions that support tactile imagery independent of the location where a stimulus was imagined. Secondly, we hypothesized that early somatosensory cortices, in particular SI, would be somatotopically activated during MI. To this end we tested for differences in SI activation between perception and imagery in relation to the hierarchical organization of the activated SI subregions.

## Materials and Methods

### Participants

Nineteen healthy, right-handed volunteers (mean age: 25.7 ± 3.8; 14 females) without any neurological or psychiatric disorder completed the study after giving written informed consent. This study was carried out in accordance with the recommendations and the approval of the Ethics Committee of the Freie Universität Berlin. All subjects gave written informed consent in accordance with the Declaration of Helsinki.

### Experimental Paradigm

**FIGURE 1:**
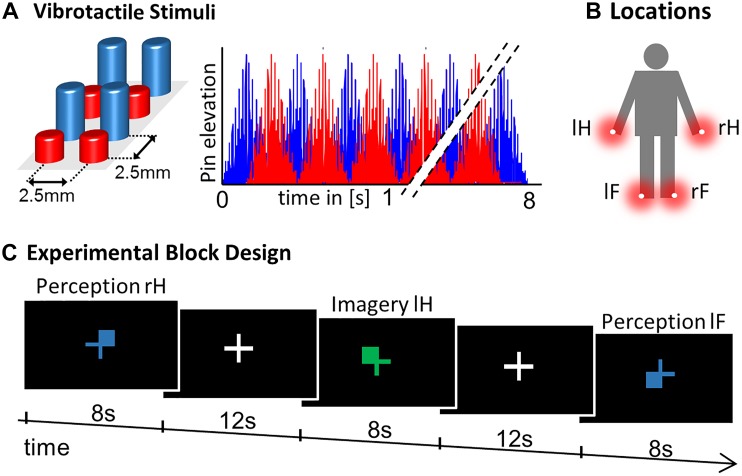
Experimental Design. **(A)** Vibrotactile stimuli were presented on a 2 x 4 pin Braille-like display. **(B)** Stimulation modules were attached to four body locations: right hand thumb (rH), left hand thumb (lH), right foot big toe (rF), and left foot big toe (lF). **(C)** The experimental paradigm constituted a block design where a visual color cue indicated where to imagine vibration in the four IMAGERY conditions. For the PERCEPTION condition the visual display was matched, where blue/green indicated PERCEPTION/IMAGERY (randomized across subjects). Each trial lasted 8 s and was followed by a 12 s inter-trial interval. Each condition was repeated three times in each of the three experimental runs, supplemented with six null-events (8 s fixation) per run.

The experimental paradigm constituted a 2 × 4 Design with factors CONDITON: perception, imagery and LOCATION: left hand thumb (lH), right hand thumb (rH), left foot big toe (lF), right foot big toe (rF). Vibrotactile stimulation was delivered using 8-dot piezoelectric Braille-like displays (2 × 4 matrix with 2.5 mm spacing) attached to the four body locations, controlled by a programmable stimulator (Piezostimulator, QuaeroSys, St. Johann, Germany). The vibrotactile stimuli were designed to elicit minimal tickling sensation and maximal stimulation intensity to assure a clear percept at fingers and toes. For 8 s a 30 Hz sinusoidal carrier signal was amplitude-modulated with a 2 Hz sinusoidal, with alternating elevation (to maximize stimulation efficiency) of the display’s four rows (Figure 1). Similar vibrotactile stimuli in the flutter range have been used in the study of perceptual and higher order cognitive functions in human and monkey research (Romo et al., 1999; Preuschhof et al., 2006; Spitzer et al., 2010; Schmidt et al., 2017), due to their well characterized, directly stimulus driven processing in SI neurons (Mountcastle et al., 1990; Hernández et al., 1997). For visual guidance through the experiment, visual cues were presented as fixation cross, which changed its color to blue/green to indicate the PERCEPTION/IMAGERY condition, where color-assignment was randomized across participants. Cues indicated where to imagine vibration or where stimulation occurred (upper left, lH; upper right, rH; lower left, lF; lower right, rF; see Figure 1) to match visual sensory stimulation between conditions. During null-events the fixation-cross remained on the screen and no stimulus was applied or imagined. While mounting subjects in the scanner, they were familiarized with the stimuli to enable imagery.

Each of three experimental runs comprised of 24 trials, corresponding to three trials per condition complemented with 6 null-events. The maximum amount of scanning time and thereby the amount of repetitions per trial type was limited due to the demanding nature of the MI tasks. To compensate for the relatively low amount of trials, we applied a block design including null-events to maximize signal changes between conditions. The order of trials was randomized and trials were intermitted by a fixed inter-trial interval of 12 s, which allowed the participants to prepare for the next trial, while no specific preparatory cue was given ahead of each trial. Stimulus presentation was controlled using custom MATLAB code (The MathWorks, MA) and the Cogent 2000 Toolbox (developed by John Romaya at the LON at the Wellcome Department of Imaging Neuroscience). Visual cues were presented on a screen that was visible from the scanner via a mirror system attached to the head coil.

### fMRI Data Acquisition

MRI data was acquired in 3 runs of 10.5 min on a 3T TIM Trio (Siemens) at the Center for Cognitive Neuroscience Berlin (CCNB). 310 functional images were recorded per run as T2^∗^-weighted gradient-echo EPI: 37 slices; interleaved order; whole brain; TR = 2000 ms; TE = 30 ms; 3 × 3 × 3 mm^3^ voxel; flip angle = 70°; 64 × 64 matrix. Additionally, a T1-weighted MPRAGE with 176 sagittal slices, TR = 1900, TE = 2.52 ms; 1 × 1 × 1 mm^3^ voxel was acquired.

### fMRI Data Analysis

Functional magnetic resonance imaging data were pre-processed with SPM8 (Wellcome Trust Centre for Neuroimaging, Institute for Neurology, University College London, United Kingdom). To minimize movement-induced image artifacts each data set was realigned to its mean image. Next, EPI images were normalized to MNI space using unified segmentation (as implemented in SPM8) and re-interpolated to 2 × 2 × 2 mm^3^ voxel size. Data was smoothed with an 8 mm FWHM Gaussian kernel. Smoothing of data for the Conjunction analyses and contrasts between PERCEPTION > IMAGERY was limited to 5 mm FWHM to preserve a high degree of regional specificity in the group level analysis.

Statistical analysis was performed according to a standard general linear model (GLM) approach. The first level design was specified to model the 8 task-conditions as independent regressors, the six null-events were split into two separate regressors (to allow independent baseline contrasts in the conjunction analysis) and a constant for each run. To test for activation clusters shared by PERCEPTION and IMAGERY we computed first-level contrasts of task-condition against null-events, with independent null-event regressors for the PERCEPTION and IMAGERY contrasts. The resulting contrast images were forwarded to a second level flexible-factorial design. We computed four conjunction analyses between perception > null-events and imagery > null-events contrast images, individually for each body location. Conjunction analyses were evaluated against the conjunction null hypothesis as implemented in SPM (Friston et al., 2005; Nichols et al., 2005).

All reported coordinates correspond to MNI space. The Anatomy Toolbox was used to establish cytoarchitectonical references where possible (Geyer et al., 1999, 2000; Grefkes et al., 2001; Eickhoff et al., 2005, 2006, 2007). Activation clusters are reported at *p* < 0.05, family wise error (FWE) corrected at the voxel level, with a cluster size threshold of 22 voxel, corresponding to the cluster size threshold of false discovery rate correction. Thresholded statistical parametric maps were rendered on a standard 3D brain template using MRIcron (by Chris Rorden; Version 6 6 2013).

To extract contrast estimates we used the activation clusters of the PERCEPTION conditions and masked them with SI subregion specific anatomical masks of the Anatomy Toolbox.

## Results

### The Brain Network Supporting Tactile Imagery

**FIGURE 2:**
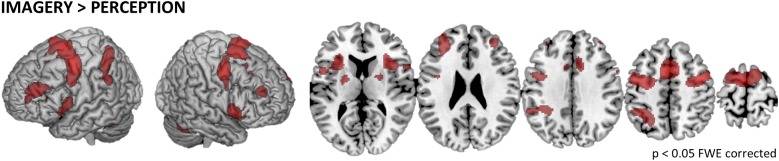
General imagery related brain activity. To depict the network of brain regions that support tactile imagery independent of content, we computed the main effect of IMAGERY > PERCEPTION. The identified network is composed of areas that are known to be modulated by general task demands such as the SMA and the preMC, which were recently associated with rehearsal processes (Fegen et al., 2015) and the medial frontal gyrus. Furthermore, activity in bilateral IFG was found, which is well known for its involvement in the processing of vibrotactile stimuli. Finally, we found left-lateralized activation in the IPS.

To test what brain regions are related to the imagery process independent of the exact location of MI, we computed the contrast IMAGERY > PERCEPTION, pooled over conditions (Figure 2). The depicted network reflects task demands of generating a mental representation from memory. The network spans regions of the task-positive network, specifically, supplementary motor areas (SMA) and frontal eye fields (FEF)/premotor cortex (preMC). Further we found bilateral medial frontal gyrus and bilateral inferior frontal gyrus (IFG). Activation in the intraparietal sulcus (IPS) was left-lateralized (Table 1).

**Table 1:** Brain activation during tactile MI.

		Peak MNI coordinates			
Anatomical region	Cluster	*X*	*Y*	*Z*	*t*-score
**IMAGERY > PERCEPTION (collapsed across conditions, *p* < 0.05 FWE corrected)**					

SMA	4913	0	2	58	11.10
Left preMC		−34	−4	60	10.00
Right preMC	947	42	−2	52	9.67
Let IPS	1434	−32	−44	42	8.88
Right insula/striatum	1202	34	15	8	8.33
Left insula	845	−30	20	8	8.07
Right cerebellum	330	38	−54	−32	7.72
Left MFG	572	−30	42	24	7.47
Left cerebellum	102	−32	−54	−32	6.82
Right MFG	155	36	44	28	5.90
Left striatum	266	−24	−2	10	5.87
Right striatum	156	20	−4	20	5.80

**PERCEPTION > other PERCEPTION conditions (*p* < 0.05 FWE corrected)**					

**Left hand finger (lH)**					
Right SI	2431	58	−16	46	13.97
Right SII		50	−18	18	13.19
**Right hand finger (rH)**					
Left SI	831	−56	−20	50	15.56
Left SII	619	−48	−20	18	12.46
**Left foot toe (lF)**					
Right SI	154	14	−44	74	7.37
Right SII	35	32	−22	14	6.30
**Right foot toe (rF)**					
Left SI	268	−14	−44	68	8.26

**IMAGERY > other IMAGERY conditions (*p* < 0.001 uncorrected)**					

**Left hand finger (lH)**					
Right SII	58	50	−18	18	5.11
Right SI	150	36	−38	52	4.03
	67	40	−26	40	3.79
**Right hand finger (rH)**					
Left SI	2	−36	−30	42	3.58
	43	−50	−32	54	3.56
	5	−30	−40	58	3.38
Left SII	1	−48	−20	18	3.25
**Left foot toe (lF)**					
Right SI	1	12	−49	68	3.40
**Right foot toe (rF)**					
Left SI	23	−12	−42	66	5.56
	15	−16	−40	70	5.15
	3	−20	−42	64	3.64
	11	−44	−22	60	3.52
Left SII	48	−34	−20	18	4.75
	13	−34	−12	10	4.05
	38	−48	−30	20	3.92
Right SI	19	48	−22	60	3.75

**Conjunction (PERCEPTION > Null) & (IMAGERY > Null) (*p* < 0.05 FWE corrected)**					

**Left hand finger (lH)**					
Right SII	86	62	−18	26	7.50
Right SI	318	34	−42	46	7.09
Left SII	64	−60	−24	24	6.72
Right insula	40	46	0	4	6.41
Right insula	36	52	10	14	6.18
**Right hand finger (rH)**					
Left SII	606	−56	−24	26	8.02
Left SI		−52	−34	52	7.88
**Left foot toe (lF)** *p* < 0.001 (uncorrected)					
right SII	202	62	−20	26	5.99
Left SII	209	−56	−24	28	5.75
Left SI	17	12	−50	70	5.41
**Right foot toe (rF)**					
Left SII	130	−58	−26	20	8.46
Left SI	39	−16	−46	70	6.45

*Activation clusters as displayed in Figure 2, 3. Results reported at p < 0.05 FWE corrected at the voxel level, cluster extend threshold of 22 voxel, and p < 0.001 uncorrected.*

### Somatotopic Activation During Perception and Imagery

**FIGURE 3:**
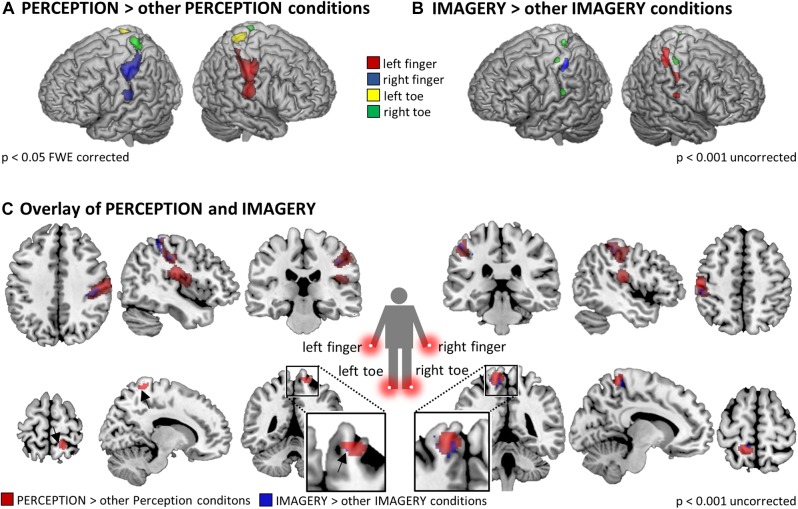
Somatotopic activation in SI and SII during **(A)** perception (*p* < 0.05 FWE corrected at the voxel level) and **(B)** imagery (*p* < 0.01 uncorrected), revealed by the contrasts of individual stimulation conditions against the three other PERCEPTION/IMAGERY conditions. As expected, the activation strength in somatosensory cortices during IMAGERY was lower than during PERCEPTION and was therefore tested on an uncorrected level following the *a priori* hypothesis of somatotopic activation in SI/SII. **(C)** Overlap of somatotopic activation in SI between PERCEPTION > other PERCEPTION conditions and IMAGERY > other IMAGERY conditions contrasts. Results displayed at *p* < 0.001 uncorrected within a SI/SII mask generated with the Anatomy Toolbox.

We first tested what regions are locus-specifically activated during perception. For each of the four PERCEPTION-conditions the first level contrast against null-events was contrasted against the three other PERCEPTION-conditions in a second level analysis. Figure 3A displays the results that reflect the well-established somatotopic organization within SI as to separate finger and foot representations and to reflect activation in SI contralateral to the body side of stimulation.

When testing in the same way for imagery related activity, namely by contrasting each of the four IMAGERY conditions against the three other IMAGERY conditions, we found weaker activation than in the PERCEPTION condition. Following our *a priori* hypothesis, we found somatotopic activation within a bilateral SI/SII mask, however, at an uncorrected significance level of *p* < 0.001. These contrasts show a shift of activation toward more posterior aspects of SI (Figure 3). This effect is apparent for left and right finger representations, while the less pronounced activation in the toe-representing regions do not allow to observe such a shift. To test if the IMAGERY activation clusters overlap somatotopically with the activation found in the PERCEPTION condition, we computed overlays between the PERCEPTION > other PERCEPTION condition contrasts and IMAGERY > other IMAGERY condition contrasts in Figure 3C.

**FIGURE 4:**
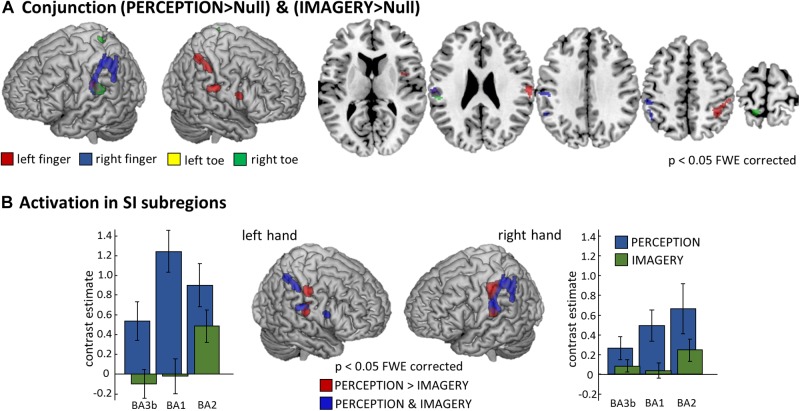
Comparison of perception and imagery. **(A)** Overlap in activation between perception and imagery identified by body-locus-specific conjunction analyses against conjunction null hypothesis (Friston et al., 2005), confirming that MI indeed recruits SI content-specifically. All results displayed at *p* < 0.05 FWE corrected at the voxel level, cluster extend threshold of 22 voxel; Note: The conjunction analysis for the right foot toe displayed also SI and SII activation however only at a threshold of *p* < 0.001 uncorrected, corresponding activation clusters are reported in Table 1. **(B)** To test what areas activate more during the PERCEPTION than during the IMAGERY condition, we computed the corresponding contrast for the left and right hand conditions and identified the hierarchically lower SI subregions (BA3b and BA1) to be stronger activated during PERCEPTION. Those SI areas that are activated in both conditions – as depicted by the conjunction analysis – are the more posterior portions of SI, corresponding to the hierarchically higher subarea BA2. To display the differences in activation level across SI subregions, we extracted first-level contrast estimates for the PERCEPTION and IMAGERY conditions contrasted against implicit baseline. These demonstrate that BA3b and BA1 do not show activation levels exceeding baseline, and that activation levels in BA2 are higher in the PERCEPTION condition than in the IMAGERY condition.

To formally test for overlapping activation between PERCEPTION and IMAGERY, we computed locus specific conjunction analyses between PERCEPTION and IMAGERY contrasts against null-events (Figure 4A). The conjunction for lH, and rH revealed clusters within SI (*p* < 0.05 FWE corrected). Inspecting the conjunction analyses for the left and right foot toe at *p* < 0.001 uncorrected also revealed a contralateral cluster in the SI foot region (Table 1), demonstrating a content-specific overlap of primary cortices activated during perception and imagery.

The well-pronounced activation clusters of the lH and rH conditions allowed us to test for their specificity within SI subregions. To this end, we tested what aspects of SI were activated more strongly for PERCEPTION than for IMAGERY. The results are compared to clusters activated in both conditions, as revealed by the conjunction analyses. Figure 4B shows that BA1 (anterior aspects of the post-central gyrus) is activated mainly during PERCEPTION. The activation cluster revealed by the conjunction PERCEPTION & IMAGERY was limited to the posterior portion of SI, matching the probability maps of BA2 according to the Anatomy Toolbox.

## Discussion

In this study, we found evidence for somatotopic recruitment of SI when participants imagined vibrotactile stimulation at different body locations. The different subprocesses of MI are difficult to dissect and it is problematic to empirically distinguish between the allocation of attention and the construction process of generating a mental content representation. The contrast IMAGERY > PERCEPTION tests for activation related to the mental construction process, irrespective of the exact content of imagery. It revealed a network of regions similar to task-positive, attention-related networks (Figure 2), comprising FEFs, SMA, and the left IPS. When testing for imagery induced activation, specific to the location where a vibratory stimulus was imagined we found somatotopic recruitment of SI (Figure 3B,C, 4A). Previous work in the visual modality interpreted retinotopic recruitment of visual cortices as evidence that our brains produce mental images using *depictive* rather than *symbolic* codes. This reasoning was mainly based on data from the visual modality. The data presented here could be interpreted along the same line of thought and thus provides a perspective that bridges modalities.

As expected, finger stimulation activated larger clusters of voxels than stimulation of toes, as it is well established that more cortical surface represents fingers than toes (Martuzzi et al., 2014). During MI of stimulation on the fingers we found well pronounced activation clusters in SI, while the activation during the MI of stimulation on the toes displayed only relatively weak activation. Nevertheless, we found activation, at *p* < 0.001 uncorrected, within SI toe representations to overlap with the activation clusters during perception (Compare Figure 3). The activation clusters found for finger regions allowed further investigation of the imagery-induced SI activation. Activation during IMAGERY was limited to perceptually activated SI subregion BA2, as revealed by the conjunction analysis between PERCEPTION and IMAGERY (Figure 4), while PERCEPTION elucidated activation spanning all SI subareas.

Finding activation only in the hierarchically highest SI subregion during IMAGERY is in line with the suggestion that lower order regions are only activated if fine-grained detailed mental images are generated (Kosslyn and Thompson, 2003). In the study at hand, only simple vibrations were imagined that did not necessitate to mentally represent fine-grained sensory details of a spatial layout. It can be argued that MI therefore did not necessitate an activation of subregions of lower hierarchical order.

### Sensory Recruitment in the Somatosensory System

Besides the study of MI, working memory (WM) studies deal with the question of what brain regions are activated to represent mental content. Actually both MI and WM are defined as the representation and manipulation of information in the absence of perceptual stimulation (Tong, 2013). The influential multi-component model of WM proposed by Baddeley suggested a *visuospatial sketchpad* as a type of *blackboard* or *buffer* on which visual information is stored (Baddeley and Hitch, 1974). Major aspects of such *sketchpad*-like representations are thought to be realized by visual cortices. So called *sensory recruitment* models of WM (Pasternak and Greenlee, 2005) emerged from observations of delay-activity in sensory regions and were well compatible with reports from the study of MI about re-activation of perceptual regions during mental reconstruction across all modalities (McNorgan, 2012; Tong, 2013; Schmidt et al., 2014). Also the MI literature emphasized the importance of topographically organized early sensory cortices for implementing a type of *visual buffer* (Kosslyn, 2005). Kosslyn compares it’s function to a *pegboard*, where different types of local information are represented topographically.

Multiple recent fMRI-WM studies using multivariate pattern analysis (MVPA) have provided support for sensory recruitment models by demonstrating that visual stimulus features can be decoded from visual cortices during WM (Christophel et al., 2015; Lee and Baker, 2016). These findings have lead to the view that sensory regions implement a memory buffer for all types of visual information (Albers et al., 2013; D’Esposito and Postle, 2015), which could consecutively also being speculated to realize the representation of mental content generated during MI.

In the tactile modality, delayed EEG activity over somatosensory regions during WM retention periods indicated distinct modality specific activation in somatosensory regions (Katus and Eimer, 2015; Katus et al., 2015). Results of a recent tactile fMRI MVPA study point in the direction that it is not primarily sensory regions that code mental contents (Schmidt and Blankenburg, 2018). This study revealed that hierarchically higher, modality-independent regions retained information throughout a 12 s WM delay phase, when participants memorized the spatial layout of a Braille-like stimulus. In contrast, somatosensory cortex was found to represent stimulus information only briefly during an early, potentially stimulus encoding, phase. Another tactile working memory study tested for the retention of the frequency of vibrotactile stimuli (Schmidt et al., 2017). Parametric multivariate codes of working memory content were found rather in prefrontal than in sensory regions, as suggested by previous electrophysiological studies (Romo et al., 1999; Spitzer et al., 2010). In sum, WM and MI studies do not allow a final conclusion about what aspects of mental images, or mental representation in general, are represented in somatosensory cortices. Results from research on visual MI and WM suggest that the levels of abstraction and sensory-detail determine whether lower order hierarchical sensory regions are recruited during MI and WM.

Here, we used simple vibratory stimuli that delivered standardized, vibratory stimulation to elicit a clear percept. In contrast to an earlier tactile MI study (Schmidt et al., 2014), the applied stimuli did not contain any relevant spatial information. Consequently, the employed task did not necessitate to generate mental images with fine-grained spatial sensory details. This might elucidate why in the current study not all parts of SI are recruited during MI, but only the higher subregion BA2 gets top-down recruited during MI. Our results could therefore also be interpreted along the view that it is the degree of sensory vividness that determines to what degree sensory regions are recruited during MI (Kosslyn and Thompson, 2003). Specifically, the more abstract information of a mental representation is represented less activation will be found in low order sensory regions. Testing this suggestion will require studies that ideally include trial-by-trial based assessments of MIs vividness. Further, different types of tactile stimuli are required to understand what stimulus features determine how these are represented in lower sensory regions. Finally, research might focus on the distinction between stimulus information being represented as categorical information, e.g., after intensive training and naming of stimuli, or rather in sensory formats as during perception.

Similarly as in previous MI studies in different modalities (McNorgan, 2012), we found the activation strength in sensory cortices to be smaller during MI than the activation during perception (Figure 4B). While vibratory stimuli have been intensively used in human WM studies (Auksztulewicz et al., 2011; Spitzer and Blankenburg, 2011; Spitzer et al., 2014), it remains an open question in how far the MI of such stimuli reaches a mental vividness to necessitate recruitment of lowest level sensory regions, which might be higher for other types of tactile stimuli. The question of what exact subprocesses are shared by imagery and perception and what subpopulations of neurons realize them is to be addressed in future research.

### The Role of Attentional Contributions to MI

The construction and maintenance of a mental image can be subdivided in different cognitive subprocesses (Kosslyn, 2005; Buckner and Carroll, 2007; Hassabis and Maguire, 2009). These include the access of long-term memory and the re-activation of a mental content by making it available for conscious processing. Most of these processes operate closely entangled with attentional mechanisms. It has become a debate whether it is possible to dissociate attentional mechanisms from neuronal processes that reflect the mere representation of a mental content, or the “pure” mental image. This challenge has been discussed in different process models of MI (Kosslyn, 2005) and it has been argued that it is not meaningful to consider them as independent psychological constructs (Gazzaley and Nobre, 2012).

Within the WM literature it was suggested to dissociate the representation of mental content from so called *cognitive control* mechanisms (Riggall and Postle, 2012; D’Esposito and Postle, 2015). It is argued that activation in task-positive networks, including the posterior parietal regions, rather reflect the *focus of attention* than the content of WM (Postle, 2015). However, it appears equally plausible to consider the content of MI (the mental image as such) as an attentional mechanism of re-activating long-term memory. Some authors even term imagery processes *reflective attention* (Chun and Johnson, 2011). More specifically, the mental representation of stimulus features might recruit attentional mechanisms that elicit activation patterns dissociable between stimuli, while not directly reflecting the stimulus properties as such. Puckett et al. (2017) recently demonstrated with high-field fMRI that attention modulates neuronal responses to tactile stimuli in a somatotopic fashion. In how far the neuronal populations modulated by attention during perception overlap with the neuronal populations activated by MI remains a central question for future MI studies. Our study will hopefully inspire high-field fMRI studies with their improved spatial resolution to map subject-specific attentional and imagery related somatotopic maps, possibly even in the subregions of SI.

Our study is limited with regards to clarifying the contributions of spatial attention to the imagery process. To limit the complexity of the study design, we did not include an attentional control condition to assess the effects of spatial attention toward a body location without imagination. Such an extension in future studies could reveal how spatial attention and MI differ in their neuronal implementation.

Besides the activation in SI it is most likely that multiple mental codes jointly represent mental content in distributed interacting cortical networks (Schlegel et al., 2013; Larocque et al., 2014; Lewis-Peacock et al., 2015; Postle, 2015; Lee and Baker, 2016). While much neuroimaging work on MI is focused on sensory cortices, there is a demand for conceptual improvements in the definition of the individual processes and psychological constructs that contribute to the MI process, particularly refined definitions of attentional contributions. Until then, it is difficult to assign clear functional labels to all the regions involved in MI. Here, we computed the main effect of IMAGERY versus PERCEPTION. This contrast reveals a distributed attention-related network of regions. The individual functional contribution of these regions remains an open question.

### Mental Imagery and Predictive Brain Mechanisms

Within the last few decades, the predictive coding (PC) framework has been promoted, most famously in the work of Karl Friston (Friston, 2009, 2010; Friston and Kiebel, 2009). This theory of global brain function has gained notable popularity beyond the neuroscientific community (Hohwy, 2014). Until now, most work on predictive mechanisms has been carried out in the context of action and perception. Very recently, however, Parr and Friston (2017) also proposed how WM might fit into this framework. They suggest WM as a process of evidence accumulation, with the main purpose of optimal policy selection for acting within the environment. This idea could be applied to the domain of MI in a very similar way.

The basic assertion of PC is that our brains continuously generate predictions about future sensory events. These predictions rely on the interaction between bottom-up and top-down signaling within the cortical hierarchy. Neuronal signals coding sensory predictions are propagated through the hierarchy via top-down connections. Sensory signals that reach the cortex from the sensory organs are propagated via bottom-up connections. If accurate predictions are generated, they match the sensory input signals. If predictions do not match the bottom-up signals, a prediction error is generated. This error signal is used to update the generative model by backpropagation to higher-order regions. The continuous influx of information from our senses requires an equal continuity of predictions. The temporary retention of information during WM and MI has to act against this continual flow of new information. Somehow, the overwriting of information as a result of the continuous sensory influx needs to be overcome in order to temporarily retain information about a specific content. Since hierarchical processing is, however, an evolutionarily old and computationally efficient principle, it is likely that the retention of mental content, as necessary for MI, is realized within the constraints of these processing principles, rather than independently of them. Depending on the level of abstraction in which a mental image is generated, an internally generated top-down signal will generate prediction errors at corresponding hierarchical levels. The energy consuming process of error generation could be the neuronal correlate of the BOLD signal changes as found in the study at hand. As this activation is driven by top-down signals and generated by different neuronal populations than bottom-up driven activation, this could also explain the difference in activation strength between imagery and perception.

Different MI and WM tasks require mental representations with different levels of detail. More abstract mental images will elicit such signals on hierarchically higher brain regions, while those mental images that have vivid sensory details will induce activation in lower order sensory regions. This PC perspective on MI can explain the different findings on sensory recruitment and explain the data from visual MI that more vivid mental images activate regions lower in the hierarchy (Kosslyn and Thompson, 2003). The tactile MI study at hand supports this principle. While imagery of a simple stimulus revealed only the hierarchically higher SI subregion BA2, the imagery of fine-grained layout information in our earlier tactile MI study (Schmidt et al., 2014) activated the hierarchically lowest cortical regions BA1 and BA3b.

## Summary and Outlook

In sum, we found imagery-induced activation within SI in a tactile MI task. Finding only the hierarchically highest SI subregion BA2 activated motivates to speculate that it is the amount of sensory vividness or sensory details of a mental image that determines which SI subregions are recruited. Taking findings from working memory studies into consideration, these findings can be well explained by top-down recruitment within the cortical hierarchy and might be implemented within neuronal circuitries of predictive brain mechanisms.

## Author Contributions

TTS and FB designed the study and analyzed the data. TTS conducted the research. TTS wrote the manuscript with contributions of FB.

## Conflict of Interest Statement

The authors declare that the research was conducted in the absence of any commercial or financial relationships that could be construed as a potential conflict of interest.
